# Bystander activation of microglia by *Brucella abortus*-infected astrocytes induces neuronal death via IL-6 trans-signaling

**DOI:** 10.3389/fimmu.2023.1343503

**Published:** 2024-01-23

**Authors:** Julia Rodríguez, Julia De Santis Arévalo, Vida A. Dennis, Ana M. Rodríguez, Guillermo H. Giambartolomei

**Affiliations:** ^1^ Instituto de Inmunología, Genética y Metabolismo (INIGEM), Consejo Nacional de Investigaciones Científicas y Técnicas (CONICET), Facultad de Farmacia y Bioquímica, Universidad de Buenos Aires, Buenos Aires, Argentina; ^2^ Center for NanoBiotechnology Research and Department of Biological Sciences, Alabama State University, Montgomery, AL, United States

**Keywords:** *Brucella abortus*, neurobrucellosis, astrocytes, microglia, phagocytosis, IL-6, trans-signaling

## Abstract

Inflammation plays a key role in the pathogenesis of neurobrucellosis where glial cell interactions are at the root of this pathological condition. In this study, we present evidence indicating that soluble factors secreted by *Brucella abortus*-infected astrocytes activate microglia to induce neuronal death. Culture supernatants (SN) from *B. abortus*-infected astrocytes induce the release of pro-inflammatory mediators and the increase of the microglial phagocytic capacity, which are two key features in the execution of live neurons by primary phagocytosis, a recently described mechanism whereby *B. abortus*-activated microglia kills neurons by phagocytosing them. IL-6 neutralization completely abrogates neuronal loss. IL-6 is solely involved in increasing the phagocytic capacity of activated microglia as induced by SN from *B. abortus*-infected astrocytes and does not participate in their inflammatory activation. Both autocrine microglia-derived and paracrine astrocyte-secreted IL-6 endow microglial cells with up-regulated phagocytic capacity that allows them to phagocytose neurons. Blocking of IL-6 signaling by soluble gp130 abrogates microglial phagocytosis and concomitant neuronal death, indicating that IL-6 activates microglia via trans-signaling. Altogether, these results demonstrate that soluble factors secreted by *B. abortus*-infected astrocytes activate microglia to induce, via IL-6 trans-signaling, the death of neurons. IL-6 signaling inhibition may thus be considered a strategy to control inflammation and CNS damage in neurobrucellosis.

## Introduction

Neurobrucellosis is an inflammatory disorder generated by the invasion of *Brucella* to the nervous system. It mostly affects the central nervous system (CNS) and has an ominous prognosis (1). Once *Brucella* spp. invades the CNS, it induces meningoencephalitis, central and peripheral demyelination, and neuritis together with neurocognitive abnormalities (2–6). *Brucella*-induced pathology in the CNS is a very complex process and it has yet many aspects to be untangled, being the reactive inflammation one of the main contributors to neuronal dysfunction (7, 8).

Astrocytes, microglia, oligodendrocytes, pericytes, and endothelial cells are part of a highly controlled microenvironment that is vital for proper neuronal function within the CNS (9). Initially, glial cells were thought to simply provide trophic support for neurons. Nowadays, it is clear that a closely intermingled neuron-glia network is a crucial requisite for adequate CNS function (10–13). Interactions between astrocytes and microglia control CNS physiology in health and disease (12–17). This molecular dialogue between microglia and astrocytes initiates early during colonization of the CNS parenchyma and plays an essential role in neuronal function (18). Yet, this reciprocal crosstalk between astrocytes and microglia may also induce CNS inflammation through the secretion of multiple cytokines and inflammatory mediators (19).

In recent years, we have demonstrated that CNS infection by *B. abortus* causes the development of a powerful inflammatory response. This response generates reactive microgliosis, astrogliosis, and cellular infiltrates (20, 21). We have also shown that after infection, glial cells secrete pro-inflammatory cytokines, chemokines, metalloproteases, and nitric oxide (NO) (8, 20, 21). Moreover, we have hypothesized that the neurocognitive symptoms associated with neurobrucellosis may be the result of neuronal damage due to the inflammatory response to infection (20, 22–24). Indeed, we have demonstrated that *B. abortus*-activated microglia induce neuronal death through primary phagocytosis (8). Nitric oxide secreted by *B. abortus*-infected microglia induced neuronal exposure of the “eat-me” signal phosphatidylserine (PS), which was sufficient to trigger the microglial milk fat globule epidermal growth factor-8 (MFG-E8)/vitronectin receptor pathway which leads to subsequent neuronal engulfment and death (8).

The role of activated microglia either as positive (16, 25) or negative regulators (26) of astrocytic pathogenic responses is well established. However, since it is becoming more apparent that inflammatory astrocytes are also able to regulate microglial activity (17, 27–29), we sought to investigate the putative role of *B. abortus*-infected astrocytes on microglial activation and the effect that this interaction might have on neuronal wellbeing. Here, we present the result of our study.

## Materials and methods

### Animals

Mice were used to obtain primary cultures of neurons, astrocytes, and microglia. BALB/c mice were provided by the School of Pharmacy and Biochemistry, University of Buenos Aires, Argentina. Interleukin-6 (IL-6) knock-out (KO) mice and C57BL/6 wild-type (WT) mice were provided by the National University of San Martín, Argentina. Mice were housed under specific pathogen-free conditions in positive-pressure cabinets and provided with sterile food and water *ad libitum*, under controlled temperature (22 ± 2°C) and artificial light (12 h cycle period). All animal procedures were performed according to the National Institute of Health (USA) rules and standards. Animal experiments were approved by the Ethics Committee of Care and Use of Laboratory Animals of the School of Medicine, University of Buenos Aires (Protocol #181/2020).

### Primary cell cultures

Cultures of cortical neurons (>95%) were generated from E16-E18 mouse forebrain fetuses, as described before (8). Briefly, meninges were removed from the brain, and cortices were dissected and treated with 0.25% trypsin-EDTA (Gibco). Cells were dissociated mechanically and plated onto glass coverslips pre-treated with poly-L-lysine (1 mg/mL) (Sigma Aldrich) at a density of 2 x 10^5^ cells/well in 24-well plates. Cultures were initially maintained with Dulbecco’s modified Eagle’s medium-F12 (DMEM-F12, Gibco) plus 10% fetal bovine serum (FBS, Gibco) for 3 h. Then, the medium was changed by a Neurobasal medium supplemented with B27 and N2 (all from Gibco). All media contained glucose, GlutaMAX (Gibco), streptomycin, and penicillin (Gibco). Astrocytes and microglia cultures (>95%) were obtained from the P1-P3 mouse forebrain following previously described procedures (20). In short, brain tissue was subjected to mechanical and enzymatic digestion with 0.25% trypsin-1mM EDTA (Gibco) along with magnetic agitation for 20 min at room temperature. The cell suspension was seeded in culture flasks previously treated with 2% w/v gelatin (Sigma Aldrich) and was grown at 37°C, 5% CO_2_ humidified incubator in DMEM with high glucose, supplemented with L-glutamine, sodium pyruvate, penicillin, streptomycin, fungizone, and 10% FBS (all from Gibco). After 2-3 weeks of culture, microglia were harvested by orbital shaking (2 h at 37°C, 180 rpm) and astrocytes were harvested by orbital shaking and subsequent trypsinization. Co-cultures of neurons/microglia were established by adding 1 x 10^5^ microglial cells on top of neuron cultures and were allowed to adhere for 18 h before treatment. Astrocytes were cultured at a density of 3.5 x 10^5^ astrocytes/well in 24-well plates.

### Bacteria

*B. abortus* S2308 was grown for 3 to 5 days in tryptic soy agar (TSA, Merck) at 37°C. An inoculum of the bacteria was suspended in sterile phosphate-buffered saline (PBS) and their number was estimated by measuring the OD600 in a spectrophotometer (Amersham Biosciences). All manipulations with viable bacteria were performed in biosafety level 3 facilities located at the INBIRS (School of Medicine, University of Buenos Aires). When indicated, *B. abortus* organisms were washed in PBS, heat-killed at 70°C for 20 min (HKBA), aliquoted, and stored at -70°C until their use to stimulate cultures. The absence of *B. abortus* viability after heat-killing was verified by the lack of bacterial growth on TSA.

### Production of astrocyte culture supernatants

Astrocyte cultures were infected with *B. abortus* at multiplicity of infection (MOI) 100 for 24 h in 0.5 mL of DMEM, supplemented with 2 mM of L-glutamine, 1 mM of sodium pyruvate, and 10% FBS (complete medium) without antibiotics. Non-infected astrocytes were cultured in the same conditions. Then, culture supernatants were collected and sterilized using a 0.22 μm filter (JetBiofil) to eliminate non-internalized bacteria, which were ultracentrifugated when mentioned (at 100,000 x g for 5 h at 4°C), aliquoted, and stored at -70°C until their use to stimulate cultures.

### Culture treatment

Neurons/microglia co-cultures or microglia cultures were stimulated for 48 h with SN from non-infected or *B. abortus*-infected astrocytes diluted in complete medium (1/2, unless otherwise stated). Untreated wells were used as a negative control. When indicated, co-cultures were treated with astrocytes SN in the presence of recombinant annexin V (200 nM; eBioscience), cyclic RGD peptide (cRGD, Cyclo(-Arg-Gly-Asp-D-Phe-Val)), cRAD (Cyclo(-Arg-Ala-Asp-D-Phe-Val)) peptides (100 μM; Bachem), or recombinant mouse IL-6 (5, 10 or 15 ng/mL; Peprotech). Also, co-cultures were pre-treated for 1 h with aminoguanidine (AG) (200 μM; Sigma Aldrich) or recombinant mouse gp130Fc chimera protein (100 ng/mL; R&D Systems), then they were treated with astrocytes SN for 48 h in the presence of the inhibitor. Neutralization experiments were performed using anti-mouse TNF-α (10 μg/mL; clone MP6-XT3; BD Pharmingen), anti-mouse IL-1β (10 μg/mL; clone B122; eBioscience), anti-mouse IL-6 (5 μg/mL; clone MP5-20F3; BD Pharmingen) monoclonal antibodies, or an isotype control (10 μg/mL; BioLegend). Culture supernatants from *B. abortus*-infected astrocytes were pre-incubated with the respective neutralizing antibodies for 1 h at 37°C before stimulating neurons/microglia co-cultures.

### Immunofluorescence and quantification of neuronal density

Neurons/microglia co-cultures were fixed with 4% paraformaldehyde (PFA) for 20 min at RT 48 h after treatment. Cells were permeabilized with 0.125% v/v Triton X-100 (Promega) and blocked with PBS 5% FBS. Neurons were labeled with anti-β-Tubulin III monoclonal antibody (1:750 dilution; clone 2G10; Sigma Aldrich) followed by Alexa Fluor 546-labeled anti-mouse IgG2a (1:200 dilution; Life Technologies Inc.). Microglia were labeled with biotinylated *Griffonia simplicifolia* isolectin-B4 (1:500 dilution; Vector Laboratories) followed by Alexa Fluor 488-labeled streptavidin (1:200 dilution; BioLegend). To dye nuclear structures, 4’,6-diamidino-2-phenylindole (DAPI, Molecular Probes) were used and nuclear morphology was analyzed to identify viable *vs*. apoptotic neurons. Images were acquired by a Nikon Eclipse Ti-E PFS microscope and analyzed using ImageJ software. Five microscopic fields per duplicate coverslip (100-150 neurons) were counted. Neuronal viability was calculated with respect to untreated controls (expressed as a percentage).

### Determination of gene expression by RT-qPCR

The total RNA of microglia cells was extracted using Quick-RNA MiniPrep Kit (Zymo Research) following the manufacturer’s instructions. cDNA was synthesized from 1 μg of total RNA using the reverse transcriptase Improm-II enzyme (Promega). Real-time quantitative PCR (RT-qPCR) was performed with the master mix FastStart Universal SYBR Green Master (ROX, Roche) in a StepOne Real-Time PCR System (Applied Biosystems). Primers used were as follows: TNF-α forward: 5’-ATGGCCTCCCTCTCATCAGT-3’, reverse: 5’-TTTGCTACGACGTGGGCTAC-3’; IL-1β forward: 5’-GCCACCTTTTGACAGTGATGAG-3’, reverse: 5’-GACAGCCCAGGTCAAAGGTT-3’; IL-6 forward: 5’-AGACAAAGCCAGAGTCCTTCAG-3’, reverse: 5’-GAGCATTGGAAATTGGGGTAGG-3’; iNOS forward: 5’-CAGCTGGGCTGTACAAACCTT-3’, reverse: 5’-CATTGGAAGTGAAGCGTTTCG-3’; β-actin forward: 5’-AACAGTCCGCCTAGAAGCAC-3’, reverse: 5’-CGTTGACATCCGTAAAGACC-3’. The amplification cycle was the following: 10 min at 95°C, 40 cycles at 95°C for 15 s, 60°C for 30 s, and 72°C for 60 s. All primer sets (Invitrogen) yielded a single amplification product by melting curve analysis. The fold change (relative expression) in gene expression was calculated using the relative quantitation method (2^−ΔΔCt^). Relative expression levels were normalized to the expression of β-actin and plotted in a log_2_ scale.

### Measurement of cytokines and nitric oxide

Mouse IL-6, IL-1β, and TNF-α were measured in culture supernatants by ELISA according to the manufacturer’s instructions (BD Pharmingen). The preexisting levels of cytokines in astrocytes SN were subtracted in order to show the specific secretion by microglia. Levels of nitric oxide (NO) in culture supernatants were evaluated by measurement of nitrite concentration using the colorimetric Griess reaction.

### Phagocytosis assays

The phagocytic capacity of microglia was evaluated by the uptake of *Escherichia coli* or negatively charged fluorescent beads. For phagocytosis assay with *E. coli*, microglial cells were washed after 24 h of culture stimulation, and *E. coli* DH5α (Invitrogen) was added for 30 min at 37°C and 5% CO_2_. Unphagocytosed bacteria were removed by washing and gentamicin treatment (100 μg/mL) for 30 min. Microglia were lysed with 0.1% v/v Triton X-100 in distilled water, and the lysates were plated on TSA and incubated overnight at 37°C. Phagocytosed bacteria were assessed by counting colony-forming units (CFU). For phagocytosis assay with fluorescent beads, after 48 h of culture stimulation, microglial cells were incubated with 0.003% w/v of 5-5.9 μm carboxyl fluorescent Nile red particles (Spherotech) for 2 h at 37°C and 5% CO_2_. Then, cells were thoroughly washed with ice-cold PBS to arrest bead internalization and fixed with 4% PFA.

### Statistical analysis

Experiments were executed at least three times using different primary cultures. Statistical analysis was performed with two-way ANOVA followed by the *post hoc* Tukey test in experiments of neuronal viability, one-way ANOVA followed by the *post hoc* Bonferroni test in experiments comparing more than two groups, or two-tailed Student’s t-test in experiments comparing two groups, using GraphPad Prism 6.0 software. Data are represented as mean ± SEM.

## Results

### Culture supernatants from *B. abortus*-infected astrocytes induce neuronal death in co-cultures of neurons/microglia

**Figure 1:**
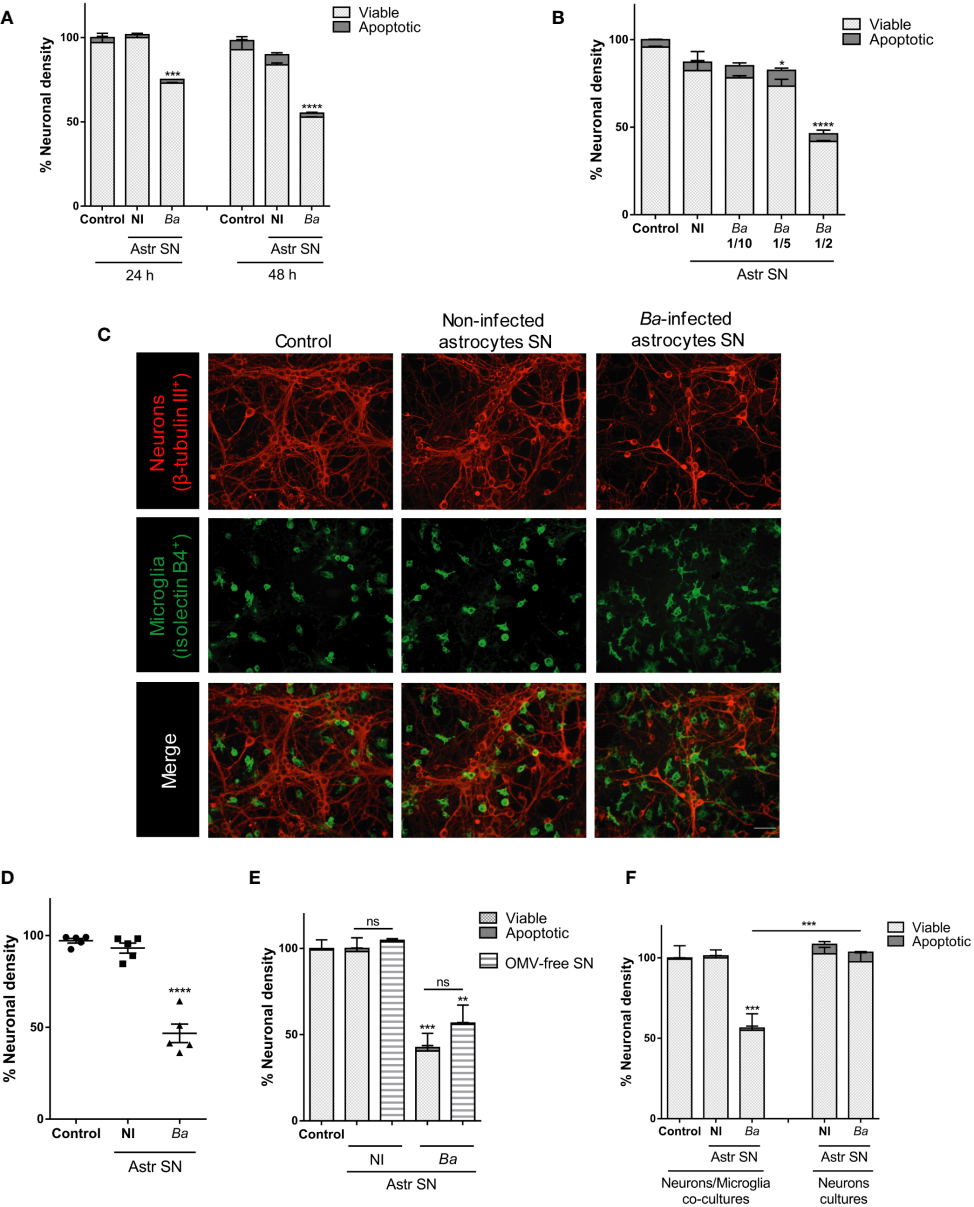
SN from *B. abortus*-infected astrocytes induce neuronal death in co-cultures of neurons/microglia. Neurons/microglia co-cultures were stimulated with SN from non-infected (NI) or *B. abortus*-infected (*Ba*) astrocytes (Astr SN) diluted in complete medium (1/2) or left untreated (control) for 24 h or 48 h **(A)**, or with different dilutions of SN from infected astrocytes for 48 h **(B)**. Representative images from neurons/microglia co-cultures showing neurons labeled with anti-β-Tubulin III antibody (red) and microglia labeled with isolectin-B4 (green). Scale bar: 50 μm **(C)**. The percentage (%) of neuronal density was evaluated in five independent experiments using neurons, microglia, and astrocytes SN from different animals **(D)**. Astrocytes SN were ultracentrifuged (OMVs-free SN) or not and used to stimulate neurons/microglia co-cultures for 48 h **(E)**. Cultures of neurons with microglia or neurons alone were treated with SN from non-infected or *B. abortus*-infected astrocytes for 48 h and neuronal density was evaluated **(F)**. The density of neurons was evaluated by fluorescence microscopy. The percentage (%) of viable and apoptotic neurons was calculated vs. control condition. Data are shown as mean ± SEM from a representative experiment of three performed, except where indicated. *p < 0.05; **p < 0.005; ***p < 0.0005; ****p < 0.0001 vs. control condition, except where indicated. Non-significant (ns).

We examined whether activation of microglia by astrocytes within the inflammatory milieu generated by *B. abortus* in the CNS promoted neuronal death. To model this scenario, *in vitro* co-cultures of neurons/microglia were stimulated with SN from *B. abortus*-infected astrocytes, and neuronal viability was determined by microscopy after 24 and 48 h of culture. In parallel, co-cultures were stimulated with SN from non-infected astrocytes or left untreated (control). The addition of SN from *B. abortus*-infected astrocytes to co-cultures of neurons/microglia induced a significant dose- and time-dependent reduction (*p* < 0.05) in the number of healthy neurons when compared with untreated controls. In all cases, the number of apoptotic neurons was low and not different from co-cultures stimulated with SN from uninfected astrocytes or unstimulated co-cultures (**Figures 1A–C**). This result did not depend on a particular co-culture or SN preparation since it was corroborated in five independent experiments using neurons, microglia, and astrocytes SN from different animals (**Figure 1D**). *Brucella* spp. releases outer-membrane vesicles (OMVs) containing lipopolysaccharide (LPS), outer membrane proteins, and other bacterial components (30). To rule out the possibility that the remaining OMVs present in SN after astrocyte infection were implicated in microglia activation, we performed ultracentrifugation of SN, as previously described (30, 31). Co-cultures were then incubated with OMV-free SN for 48 h and neuronal death was evaluated. The percentage of neuronal death was not significantly different (*p* > 0.05) between non-depleted and OMV-free SN (**Figure 1E**). Importantly, *B. abortus*-infected astrocytes SN had no direct effect on neuronal viability if microglia were not present in the culture (**Figure 1F**), indicating that SN are activating microglia to induce neuronal demise. These results indicate that secreted factors from *B. abortus*-infected astrocytes are responsible for microglia activation and concomitant neuronal death.

### SN from *B. abortus*-infected astrocytes induce microglial activation

**Figure 2:**
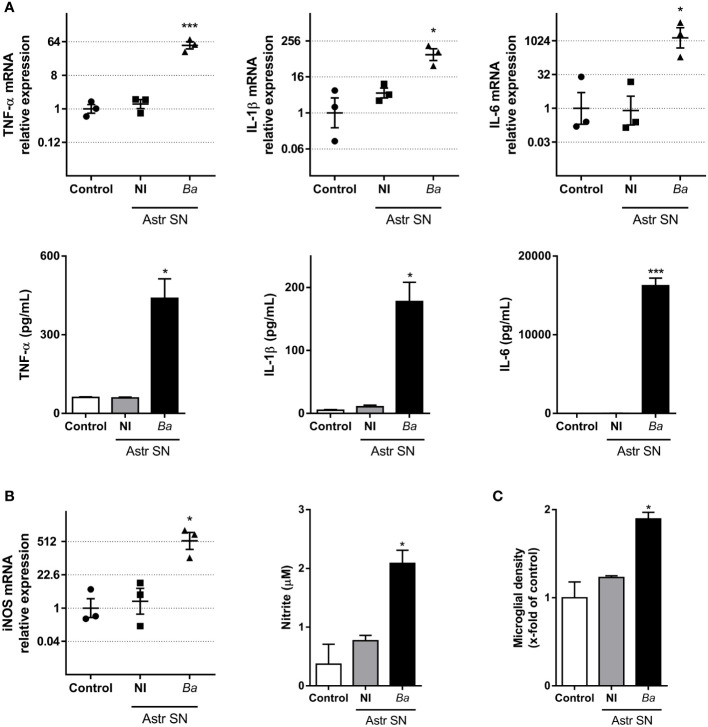
SN from *B. abortus*-infected astrocytes induce an inflammatory phenotype on microglia. Microglia were treated with SN from non-infected (NI) or *B. abortus*-infected (*Ba*) astrocytes (Astr SN) or left untreated (control) for 48 h. Gene expression of TNF-α, IL-1β, and IL-6 was analyzed by RT-qPCR in three independent experiments (plotted in a log2 scale) and cytokine secretion was measured by ELISA **(A)**. Gene expression of iNOS was determined by RT-qPCR in three independent experiments and the level of NO was evaluated by Griess reaction **(B)**. Proliferation was assessed by fluorescence microscopy **(C)**. Data are shown as mean ± SEM from a representative experiment of three performed, except where indicated. *p < 0.05; ***p < 0.0005 vs. control condition.

**Figure 3:**
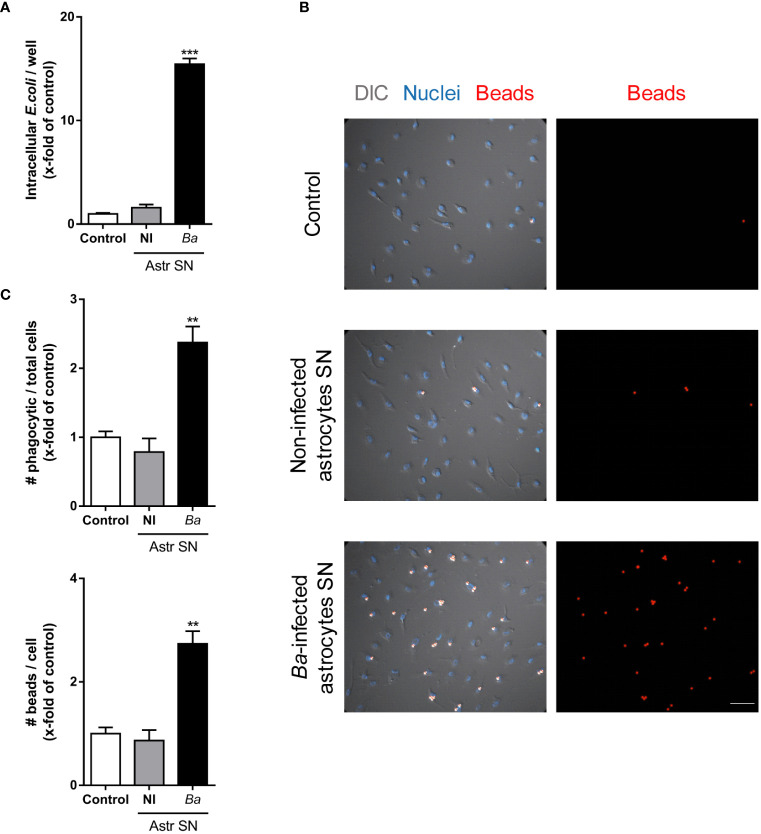
SN from *B. abortus*-infected astrocytes increase the phagocytic activity of microglia. Microglia cultures were treated with SN from non-infected (NI) or *B. abortus*-infected (*Ba*) astrocytes (Astr SN), or left untreated (control). The phagocytic activity of microglia was evaluated by two different phagocytosis assays using *E. coli* **(A)** or negatively charged fluorescent 5 μm beads **(B, C)**. Phagocytized bacteria were evaluated by intracellular CFU counting, and phagocytized beads were evaluated by fluorescence microscopy. Scale bar: 50 μm. Data are shown as mean ± SEM from a representative experiment of three performed. **p < 0.005; ***p < 0.0005 vs. control condition.

To investigate the effects that SN from *B. abortus* ***-*** infected astrocytes have on microglia, we evaluated several parameters of microglia activation. Treatment with SN from *B. abortus* ***-*** infected astrocytes induced a significant (*p* < 0.05) inflammatory activation of microglia measured as an increased gene transcription and secretion of TNF-α, IL-1β, and IL-6 (**Figure 2A**); increased gene transcription of the inducible nitric oxide (NO) synthase (iNOS) expression with concomitant NO release (**Figure 2B**); and increased microglial proliferation (**Figure 2C**). Treatment also increased the phagocytic capacity of microglia. Microglia treated with SN from *B. abortus*-infected astrocytes significantly (*p* < 0.0005) increased the phagocytosis of *E. coli* when compared with unstimulated control microglia or microglia stimulated with SN from uninfected astrocytes (**Figure 3A**). Activation of microglia caused by treatment with SN from *B. abortus* ***-*** infected astrocytes also significantly (*p* < 0.05) improved phagocytic uptake of beads, increasing both the number of phagocytic microglia and the number of beads taken per microglia (**Figures 3B, C**), when compared with microglia incubated with SN from uninfected astrocytes or unstimulated control microglia. Overall, these results indicate that SN from *B. abortus* ***-*** infected astrocytes induce an inflammatory activation of microglia with the release of pro-inflammatory mediators and the increase in their proliferation and phagocytic capacity which results in the death of neurons.

### Microglia activated by SN from *B. abortus*-infected astrocytes induce neuronal death by primary phagocytosis

**Figure 4:**
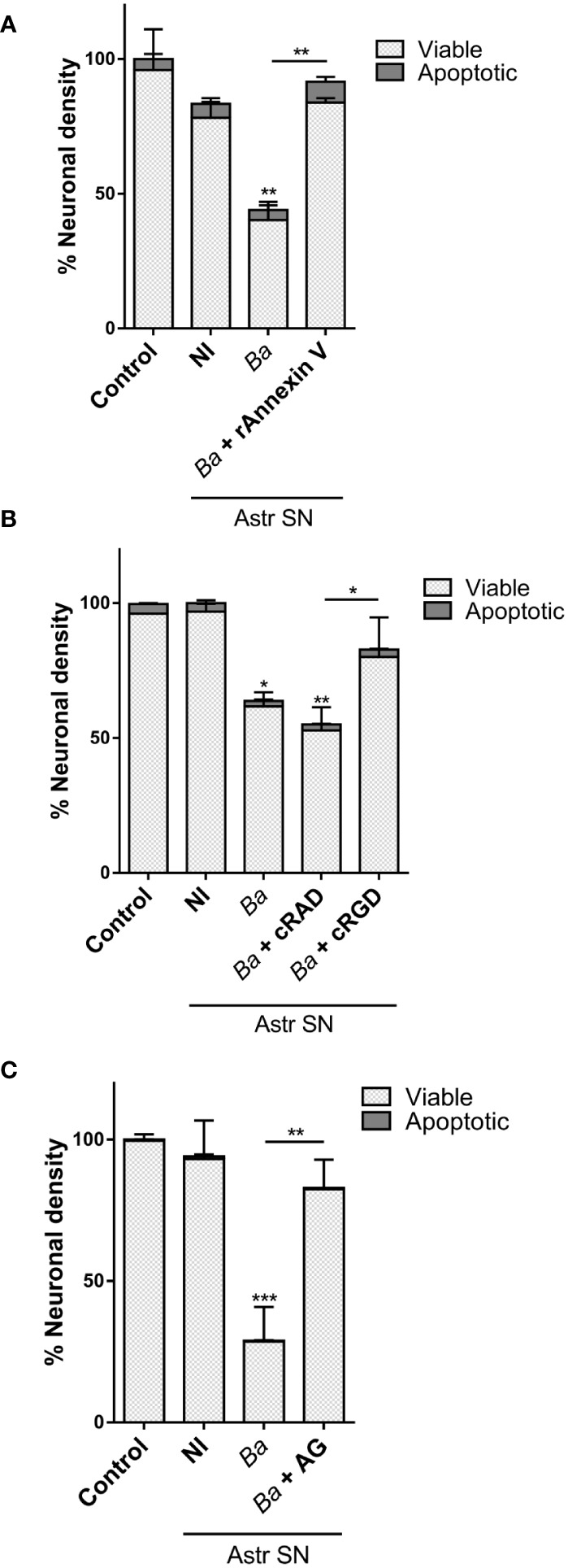
Microglia activated by *B. abortus*-infected astrocytes induce neuronal death by primary phagocytosis. Neurons/microglia co-cultures were stimulated with culture supernatants (Astr SN) from non-infected (NI) or *B. abortus*-infected (*Ba*) astrocytes for 48 h in the absence or the presence of recombinant Annexin V (rAnnexin V; 200 nM) **(A)**, the cyclic (c) peptides cRAD or cRGD (100 μM) **(B)**, or aminoguanidine (AG; 200 μM) **(C)**. Untreated co-cultures were used as control conditions (control). The density of neurons was evaluated by fluorescence microscopy. The percentage (%) of viable and apoptotic neurons was calculated vs. control. Data are shown as mean ± SEM from a representative experiment of three performed. *p < 0.05; **p < 0.005; ***p < 0.0005 vs. control condition, except where indicated.

In a previous report, we have demonstrated that *B. abortus*-infected microglia induce neuronal loss by primary phagocytosis (also named phagoptosis) of live neurons (8). The elimination of neurons relies on two simultaneous events: inflammatory signaling–specifically NO secretion–and the increased phagocytic capacity of microglia. NO secreted by *B. abortus*-activated microglia induced neuronal exposure of the “eat-me” signal PS which allows the microglial engulfment of live neurons through the vitronectin receptor, using MFG-E8 as a bridging molecule. Blocking either of the two mechanisms (NO secretion or phagocytosis) abrogates neuronal death without inhibiting microglial overall activation (8). Thus, to investigate if phagoptosis was involved in the neuronal death induced by microglia activated by SN from *B. abortus*-infected astrocytes, we first inhibited the PS/MGF-E8/vitronectin receptor pathway. Blocking the MFG-E8–neuron’s PS interaction (using recombinant annexin V) or the interaction between MFG-E8 and the microglia’s vitronectin receptor (using cRGD) completely inhibited the neuronal loss induced by microglia activated by SN from *B. abortus*-infected astrocytes (**Figures 4A, B**). As expected, the control peptide cRAD did not prevent neuronal death. Of note, in both cases (annexin V and cRGD), the number of apoptotic neurons was not higher than controls (**Figures 4A, B**), indicating that microglial phagocytosis of neurons was the cause of death rather than its consequence. Certainly, if neurons had been killed first by microglia and afterward phagocytosed by them, when inhibition of phagocytosis was performed, apoptotic neurons should have been visualized in the culture. Instead, we have found only live neurons in the cultures. Likewise, the addition of aminoguanidine, an inhibitor of both constitutive and iNOS and therefore the release of NO (8), significantly reduced (*p* < 0.005) the neuronal death induced by microglia activated by SN from *B. abortus*-infected astrocytes (**Figure 4C**). These results indicate that microglia stimulated by SN from *B. abortus*-infected astrocytes kill live neurons by primary phagocytosis.

### Neutralization of IL-6 prevents neuronal death induced by SN-activated microglia

**Figure 5:**
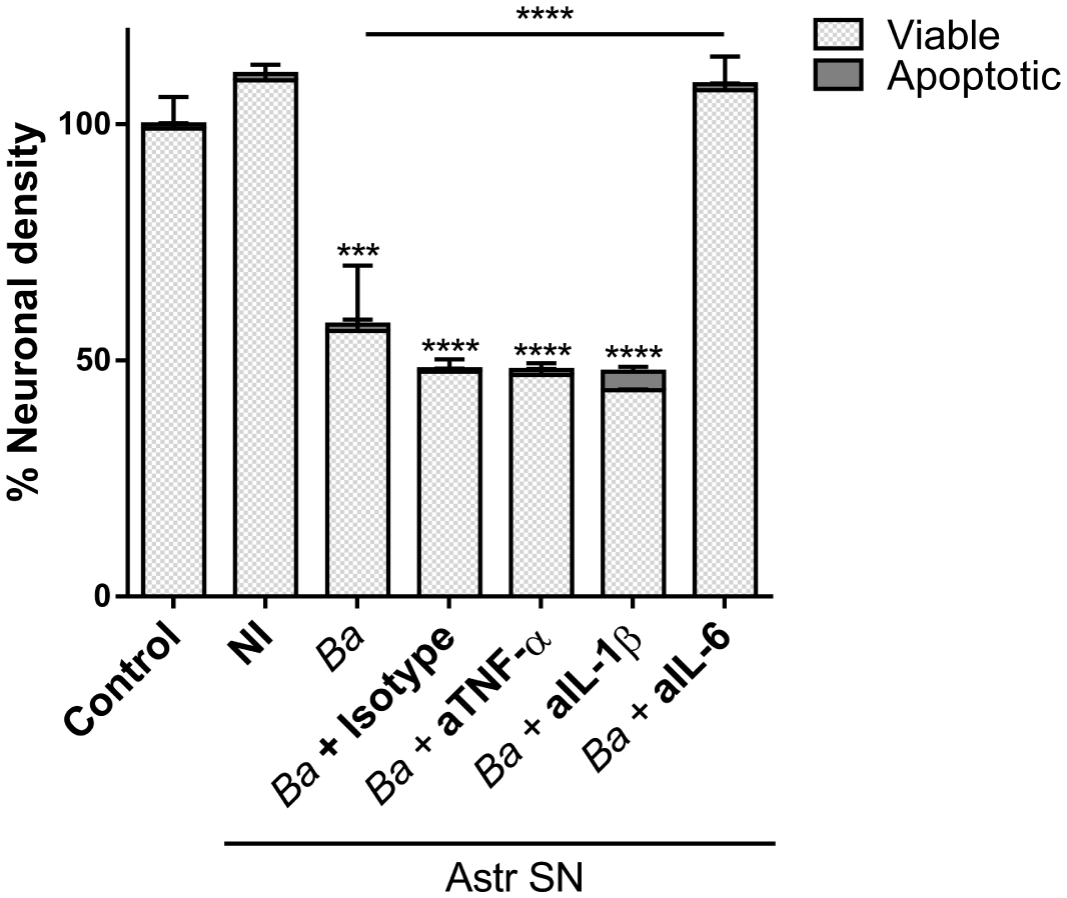
Neutralization of IL-6 prevents neuronal death induced by bystander-activated microglia. SN from non-infected (NI) or *B. abortus*-infected (*Ba*) astrocytes (Astr SN) were pre-incubated or not with anti-TNF-α (aTNF-α; 10 μg/mL), anti-IL-1β (aIL-1β; 10 μg/mL), anti-IL-6 (aIL-6; 5 μg/mL) monoclonal antibodies, or isotype control (10 μg/mL) and used to stimulate neurons/microglia co-cultures for 48 h. Untreated co-cultures were used as control conditions (control). The percentage (%) of viable and apoptotic neurons was calculated vs. control. Data are shown as mean ± SEM from a representative experiment of three performed. ***p < 0.0005; ****p < 0.0001 vs. control condition, except where indicated.

Our results indicate that soluble factors secreted by *Brucella*-infected astrocytes are involved in the activation of microglia which leads to neuronal death by primary phagocytosis. TNF-α, IL-1β, and IL-6 have been shown to be key inducers of microglial activation and regulators of microglia effector functions (32–35). Because these cytokines are secreted upon infection of astrocytes with *B. abortus* (20), we sought to investigate their role in the activation of microglia by SN from *B. abortus*-infected astrocytes. To investigate the role of IL-6, IL-1β, and TNF-α in neuronal phagoptosis induced by microglia activated by SN from *B. abortus*-infected astrocytes, we pre-incubated SN with IL-6, IL-1β, or TNF-α neutralizing antibodies before adding them to microglia/neurons co-cultures. Neutralization of IL-6 but not TNF-α or IL-1β resulted in complete abrogation of neuronal death induced by activated microglia. Isotype control antibody had no effect on primary phagocytosis of neurons induced by activated microglia (**Figure 5**). This result indicates that IL-6 is a key factor in activating microglia to induce neuronal death.

### IL-6 secreted by both astrocytes and microglia contributes to neuronal death induced by *B. abortus*-activated microglia

**Figure 6:**
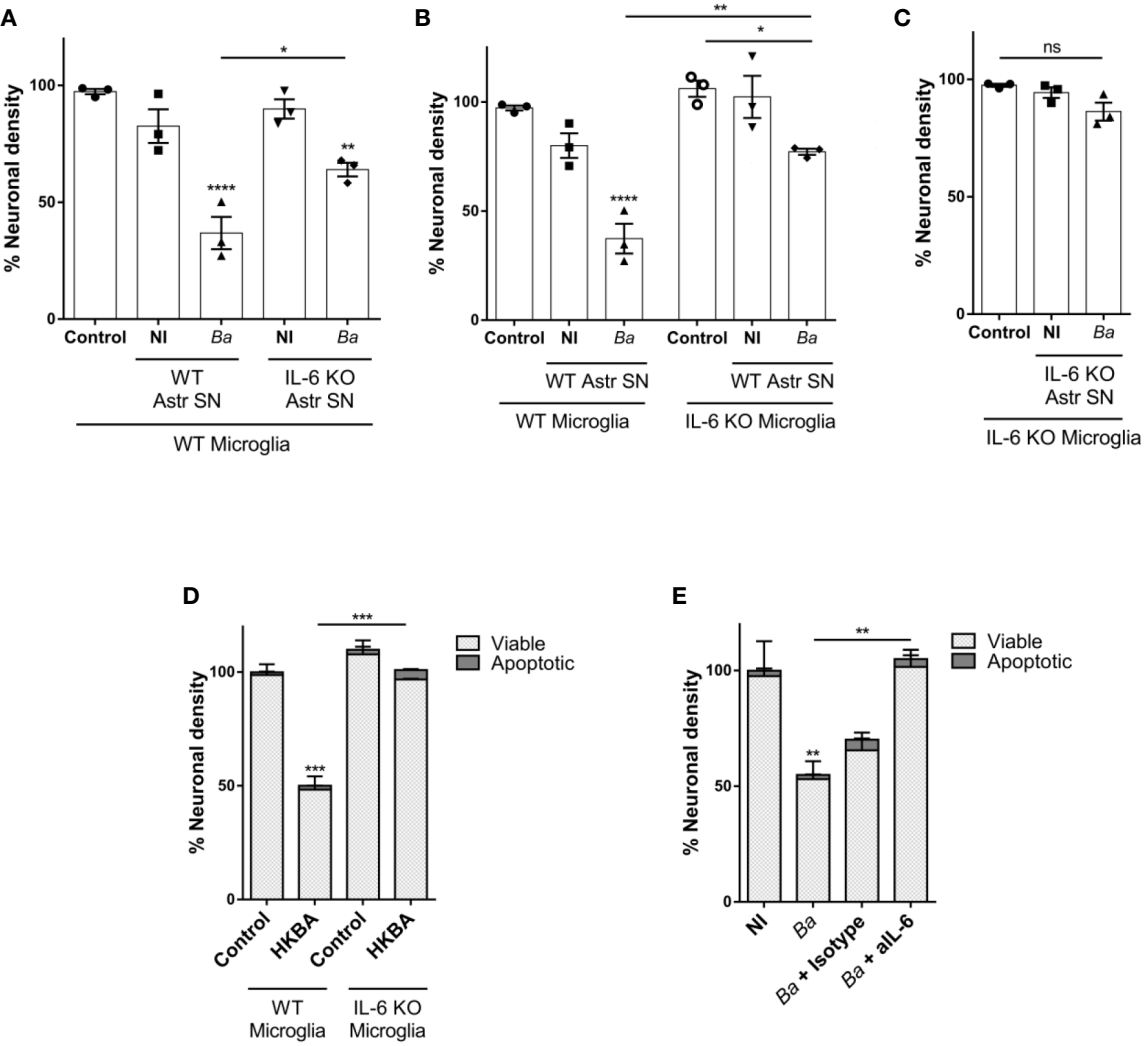
IL-6 secreted by both astrocytes and microglia contributes to neuronal death by primary phagocytosis. Neurons/microglia wild-type (WT) co-cultures were stimulated with SN from non-infected (NI) or *B. abortus*-infected (*Ba*) WT and IL-6 knock out (KO) astrocytes (Astr SN) for 48 h **(A)**. Co-cultures of neurons and WT or IL-6 KO microglia were treated with SN from non-infected (NI) or (*B*) *abortus*-infected (*Ba*) WT astrocytes for 48 h **(B)**. Co-cultures of WT neurons and IL-6 KO microglia were treated with SN from non-infected (NI) or (*B*) *abortus*-infected (*Ba*) IL-6 KO astrocytes for 48 h **(C)**. Co-cultures of neurons and WT or IL-6 KO microglia were stimulated with heat-killed *(B) abortus* (HKBA; 1x10^8^ bacteria/mL) for 48 h **(D)**. Neurons/microglia co-cultures were infected with *B. abortus* (MOI 100) in the presence of anti-IL-6 monoclonal antibody (aIL-6; 5 μg/mL) or its isotype control (5 μg/mL) for 48 h **(E)**. The percentage (%) of neuronal density is shown as mean ± SEM from three independent experiments using neurons, microglia, and astrocytes SN from different animals **(A-C)**. The percentage (%) of viable and apoptotic neurons was calculated vs. WT control (untreated) condition. Data are shown as mean ± SEM from a representative experiment of three performed **(D, E)**. *p < 0.05; **p < 0.005; ***p < 0.0005; ****p < 0.0001 vs. WT control condition, except where indicated. Non-significant (ns).

Our results (**Figure 2A** and (20)) indicate that *Brucella*-activated microglia are capable of secreting IL-6. This, together with the experimental design of the neutralization assay performed above cannot rule out a putative contribution of microglial-secreted IL-6 in the phagoptosis induced by microglia activated by SN from *B. abortus*-infected astrocytes. To decipher the source of IL-6 (microglia or astrocyte SN, or both) that drives activated microglia to kill neurons, we performed co-cultures with microglia and astrocyte SN from mice deficient in the IL-6 gene (IL-6 knock-out, KO). At first, we treated wild-type (WT) co-cultures of neurons/microglia with SN from *B. abortus*-infected WT or IL-6 KO astrocytes (**Figure 6A**). As was shown before, when SN from *B. abortus*-infected WT astrocytes were employed to activate WT microglia (▲), a significant (*p* < 0.0001) neuronal death was achieved when compared to control cultures (●). Stimulation of WT microglia with SN from *B. abortus*-infected IL-6 KO astrocytes (♦) caused a significant neuronal death compared with the control condition (●, *p* < 0.005) even though the percentage of neuronal death was significantly less (*p* < 0.05) than the one obtained with SN from WT-infected astrocytes (▲). Conversely, we performed co-cultures of neurons with WT or IL-6 KO microglia and treated them with SN from *B. abortus*-infected WT astrocytes (**Figure 6B**). Likewise, if IL-6 KO microglia were activated with SN from *B. abortus*-infected WT astrocytes (♦), the percentage of neuronal death was significantly less than the one obtained in co-cultures with WT microglia (▲, *p* < 0.005) although still significant from the untreated control co-cultures with IL-6 KO microglia (**○**, *p* < 0.05). Finally, in co-cultures where both microglia and SN from *B. abortus*-infected astrocytes were obtained from IL-6 KO mice, no neuronal death was achieved (**Figure 6C**) indicating the key role of this cytokine in mediating microglia-induced neuronal death. Indeed, the importance of IL-6 in the overall phagoptotic phenomenon was corroborated by stimulating microglia with heat-killed *B. abortus* (HKBA), a surrogate of infection (8). At variance with WT microglia, IL-6 KO microglia were unable to induce neuronal phagoptosis upon activation by *B. abortus* directly (**Figure 6D**). Also, infection of WT microglia in the presence of IL-6 neutralizing antibody completely abrogated neuronal death (**Figure 6E**). These results indicate that IL-6 from both astrocytes and microglia contributes to neuronal death and demonstrate the critical role of this cytokine in the phagoptosis of neurons induced by *B. abortus* regardless of its cellular source.

### IL-6 neutralization inhibits the phagocytic activity of microglia, but not its inflammatory activation

**Figure 7:**
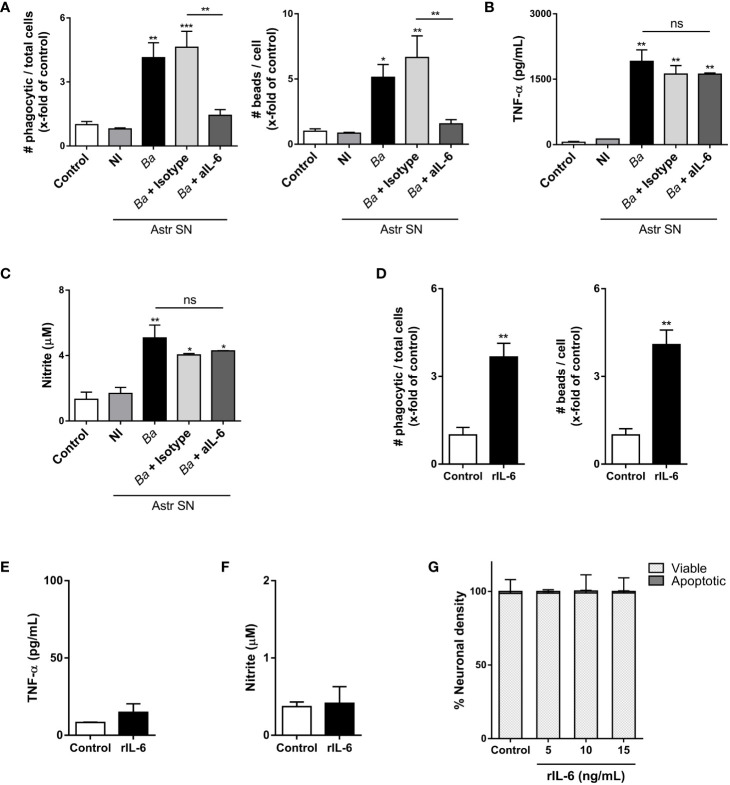
IL-6 neutralization inhibits the phagocytic activity of microglia, but not its inflammatory activation. Microglia cultures were stimulated with SN from non-infected (NI) or *B. abortus*- infected (*Ba*) astrocytes (Astr SN) in the presence of anti-IL-6 monoclonal antibody (aIL-6; 5 μg/mL) or its isotype control (5 μg/mL) for 48 (h) Untreated co-culture was used as a control condition (control). Phagocytic activity was evaluated by a phagocytosis assay with fluorescent 5 μm beads that were visualized by fluorescence microscopy **(A)**. Secretion of TNF-α **(B)** and NO **(C)** were also measured in cultured supernatants. Microglia cultures were treated with recombinant IL-6 (rIL-6; 15 ng/mL) for 48 h and phagocytic activity **(D)**, secretion of TNF-α **(E)**, and release of NO **(F)** were measured. Neurons/microglia co-cultures were treated with different concentrations of rIL-6 for 48 h, and neuronal density was evaluated. Untreated co-culture was used as a control condition (control). The percentage (%) of viable and apoptotic neurons was calculated vs. control **(G)**. Data are shown as mean ± SEM from a representative experiment of three performed. *p < 0.05; **p < 0.005; ***p < 0.0005 vs. control condition, except where indicated. Non-significant (ns).

As we have mentioned before, the elimination of neurons by primary phagocytosis relies on two simultaneous events: NO secretion (which induces the exposure of the “eat-me” signal PS on neurons) and the increased phagocytic capacity of microglia (8). To investigate in which of these two phenomena (if not in both) IL-6 is involved, we performed neutralization experiments using IL-6 neutralizing antibodies and evaluated microglia functions. Neutralization of IL-6 resulted in complete abrogation of the phagocytic ability of microglia as induced by SN from *B. abortus*-infected astrocytes, as compared to microglia treated with SN from uninfected astrocytes or untreated control ones, when the phagocytic capacity was evaluated as uptake of negatively charged microbeads (both the number of phagocytic microglia and the number of beads taken per microglia) (**Figure 7A**). Conversely, the inflammatory activation of microglia measured as TNF-α secretion; and more importantly, NO release, was not modified when SN were treated with neutralizing IL-6 antibodies (**Figures 7B, C**). Isotype control antibody has no effect in none of these phenomena. These results indicate that IL-6 increases the phagocytic activity of microglia, but it does not modify its inflammatory activation. Indeed, although recombinant IL-6 increased the microglial phagocytosis of beads (**Figure 7D**), without inducing an inflammatory response (as TNF-α and NO secretion) (**Figures 7E, F**), it did not induce neuronal loss (**Figure 7G**). Overall, these results indicate that IL-6 is necessary, but not sufficient to induce neuronal death by primary phagocytosis since it is only involved in the increased phagocytic capacity of microglial cells.

### IL-6 activates microglia via trans-signaling

**Figure 8:**
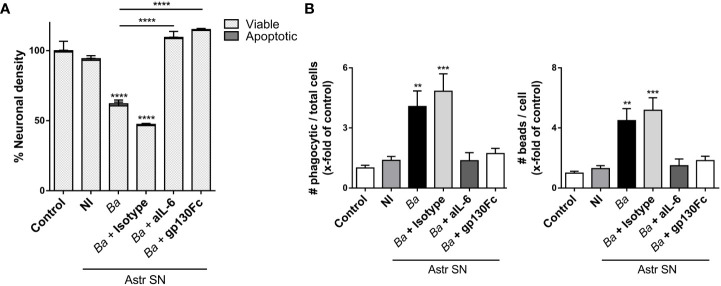
IL-6 activates microglia via trans-signaling. SN from non-infected (NI) or *B. abortus*-infected (*Ba*) astrocytes (Astr SN) were pre-incubated or not with recombinant gp130Fc (100 ng/mL), anti-IL-6 (aIL-6; 5 μg/mL) monoclonal antibody, or its isotype control (5 μg/mL) and used to stimulate neurons/microglia co-cultures **(A)** or microglia cultures **(B)** for 48 (h) Untreated cultures were used as control conditions (control). Percentage (%) of viable and apoptotic neurons was calculated vs. control **(A)**. The phagocytic activity of microglia was evaluated **(B)**. Data are shown as mean ± SEM from a representative experiment of three performed. **p < 0.005; ***p < 0.0005,****p < 0.0001 vs. control condition, except where indicated.

IL-6 may stimulate responses in a target cell in two different manners. Classical signaling involves the binding of IL-6 to the membrane-bound IL-6 receptor (IL-6R). As the IL-6R lacks intrinsic signal transduction capacity, subsequent downstream signaling takes place upon engagement of its β-receptor gp130 on the cell membrane (36, 37). IL-6R also exists as a soluble protein (sIL-6R), which forms a complex with IL-6 and stimulates cells that express gp130 with or without endogenous IL-6R expression in a mechanism known as trans-signaling (38). To complicate things further, a soluble form of gp130 (sgp130) is also formed. sgp130 acts as a decoy receptor and can inhibit trans-signaling but does not affect classical signaling (39). As neutralizing IL-6 antibodies block both classical and trans-signaling, we used a recombinant gp130-Fc chimerical protein to investigate which signaling pathway is involved in the activation of microglia. For this, neurons/microglia co-cultures were treated with SN from *B. abortus*-infected astrocytes in the presence of gp130-Fc and its effect *vis-à-vis* the effect of IL-6 neutralizing antibodies was compared. The presence of gp130-Fc completely inhibited neuronal death induced by activated microglia. Again, neutralization of IL-6 also resulted in complete abrogation of neuronal death induced by activated microglia. Isotype control antibody had no effect on primary phagocytosis of neurons induced by activated microglia (**Figure 8A**). Moreover, the presence of gp130-Fc resulted in complete abrogation of microglia´s phagocytic capacity induced by SN from *B. abortus*-infected astrocytes (**Figure 8B**). Thus, trans-signaling was involved in mediating the phagocytic activity of microglia treated with SN from *B. abortus*-infected astrocytes. These results indicate that IL-6 trans-signaling increases microglia phagocytosis, leading to neuronal death.

## Discussion

The entry of *B. abortus* to the brain parenchyma takes place within infected monocytes as a Trojan horse after peripheral inflammation of the blood-brain barrier induced by the bacterium (31, 40). In turn, these infected monocytes served as bacterial sources for *de novo* infection for astrocytes and microglia (40). Infection of glial cells has a profound impact on neurobrucellosis physiopathology, provoking deleterious consequences on astrocytes (20), the blood-brain barrier (41), and neurons (8). Although it is well recognized that when *B. abortus* infects a cell population, *Brucella-*infected cells constitute only 5-10% of all cells (42, 43); the activation of astrocytes and microglia during neurobrucellosis is widespread (20), potentially indicating that a bystander activation of non-infected glial cells could be taking place in the inflammatory milieu generated by infection. Thus, we sought to investigate the role of *B. abortus*-infected astrocytes on microglial activation and how this interaction might affect neuronal health.

Our results demonstrate that SN from *B. abortus*-infected astrocytes induce the inflammatory activation of microglia, which, in turn, execute the death of neurons. It has been well documented that in the context of infection, there exists a bystander activation of uninfected cells by inflammatory mediators released by adjacent infected cells that allow the amplification and propagation of deleterious innate immune responses in most tissues (44–46). In this context, the relevance of infection of astrocytes relies on the fact that these cells are the most abundant cellular type of the CNS, in comparison with microglia which comprise approximately 5-10% of the brain parenchyma (47). Thus, astrocytic inflammatory activation would have a profound impact on the overall innate immune activation of CNS during neurobrucellosis by activating neighboring glial cells such as microglia (48), which in turn become lethal to neurons.

SN from *B. abortus*-infected astrocytes could not directly induce neuronal death, which is similar to our previous observations using conditioned media from *B. abortus*-infected microglia (8). In both cases, we have demonstrated the requirement of the presence of and close contact between microglia and neurons for neuronal demise to occur, a condition that enables phagocytosis of live neurons by activated microglia. SN from *B. abortus* ***-*** infected astrocytes induce the overall activation of microglia with the release of pro-inflammatory mediators and the increase of their phagocytic capacity. These two features were key in the execution of live neurons by phagoptosis, a recently described mechanism whereby microglia activated by *B. abortus* kill neurons by phagocytosing them (8). Thus, the phagoptosis of neurons by *B. abortus*-activated microglia might be triggered at least in two ways: by direct *B. abortus* infection of microglia or by a bystander microglial activation through inflammatory mediators secreted by *Brucella*-infected astrocytes, vindicating primary phagocytosis of neurons as a major contributor of CNS pathology in brucellosis.

It has been described that a transgenic mouse model with astrocytes overexpressing IL-6 (GFAP-IL6) in the CNS exhibits neurodegeneration associated with learning (49) and motor impairment (50, 51). Interestingly, brain sections of these mice exhibit activation of microglia (microgliosis), which correlates with the loss of different neuronal subpopulations. However, the molecular mechanisms underlying neuronal death in these models have not been reported. Our findings describe a mechanism in which IL-6 is implicated in the neuronal death induced by microglia activated by SN from *B. abortus*-infected astrocytes. Moreover, our results demonstrate that IL-6 is a key cytokine necessary for primary phagocytosis of neurons to occur since neutralization of this cytokine completely abrogates neuronal loss. The mechanism of action, the cellular source, and the signaling pathway of IL-6 in our model merit discussion.

IL-6 is solely involved in increasing the phagocytic capacity of activated microglia as induced by SN from *B. abortus*-infected astrocytes and does not participate in their inflammatory activation, ascribing to IL-6 a direct action on the microglial phagocytic function. This is true in our neurobrucellosis model and also in other models in which IL-6 elicited transcriptomic and molecular changes that increase the phagocytic capacity of phagocytes (52, 53). Moreover, in some of these models, the IL-6-mediated increment of the phagocytic capacity of microglia also correlates with neurodegeneration (54, 55). Interestingly, IL-6 was also shown to increase the expression and the adhesion capacity of the α_v_ subunit of the vitronectin receptor (56), the receptor involved in the neuronal loss induced by *B. abortus*-activated microglia ( (8) and **Figure 4**). Our results also indicate that although necessary, IL-6 is not sufficient to induce microglia-mediated neuronal death since the concomitant release of NO by activated microglia must take place for neuronal loss to occur.

IL-6 is a major cytokine in the CNS produced by glial cells, neurons, and endothelial cells under physiological and pathological conditions (57), including neurobrucellosis (20, 41, 58). Particularly, in neurobrucelosis, infiltrating CD8^+^ T-cells (59) could also be a source of IL-6, as it has been described in multiple sclerosis (60). Despite its autocrine action on many of these IL-6-producing cells (61–63), the functions exerted by IL-6 in CNS are induced in most cases in a paracrine way on neighboring cells (27, 54, 55, 64). In this complex scenario, astrocyte-derived IL-6 seems to have a major role in microglia functions (29, 51, 55). Our results show that both autocrine microglia-derived and paracrine astrocyte-secreted IL-6 add up to endow microglial cells with up-regulated phagocytic capacity that allows them to phagocytose neurons and go along with the contention of a multicellular source of production for IL-6 in CNS to undertake both physiological and pathological functions (34, 57).

The pleiotropic functions of IL-6 are clearly depicted in CNS where it promotes anti- and pro-inflammatory outcomes (65). IL-6 also plays a critical role in the normal homeostasis of neuronal tissue stimulating neurogenesis, neuronal differentiation, and neuroprotection against tissue injury (65, 66). Under homeostatic conditions, low levels of IL-6 are detectable in the brain (66). Yet, its production increases during many pathological conditions, including neurobrucellosis (58). This IL-6 overproduction in the brain usually leads to neurodegeneration (49, 50). In fact, several authors suggest that IL-6 might be a relevant biomarker for poor prognosis in several mental pathologies such as depressive disorders (67) and schizophrenia (68), or in the use of drugs such as cannabis and ketamine (69, 70), which are conditions related to neuronal dysfunction and with similar symptoms to the ones of neurobrucellosis (3–5). In any case, classification of anti- or pro-inflammatory profiles by IL-6 on CNS is sensitive not only to its levels (low levels under healthy steady state conditions vs. high levels during pathological conditions), cellular source (neurons, astrocytes, oligodendrocytes, microglia, and endothelial cells), and targets of IL-6 itself but more important to the IL-6 signaling pathway.

Our results demonstrate that neuronal death induced by microglia activated by SN from *B. abortus*-infected astrocytes proceeds via trans-signaling and agree with the contention that neuronal damage depends on trans-signaling in the CNS, whereas classical signaling has a regenerative role in neural tissue (71). Of note, although IL-6 by itself has been reported to elicit gp130-mediated signaling in IL-6R-bearing microglial cells, these cells are also responsive to hyper-IL-6 (a recombinant chimera that mimics trans-signaling) (34, 72). Notably, in pathological states, it seems that classical signaling is not involved in the activation of microglial cells as microglia become pro-inflammatory when exposed to hyper-IL-6 *versus* IL-6 alone (73), emphasizing the different pathological consequences between classical and trans-signaling on microglia.

In fact, classical IL-6 signaling has been hypothesized to have a neuroprotective role within the CNS (74–76). Although still under debate in the field, currently available data have confirmed a context-dependent and mutual modulation of classical or trans-signaling IL-6 pathways that lead to anti- and pro-inflammatory responses (65).

Finally, it is our contention that neuronal death caused by primary phagocytosis induced by *B. abortus*-activated microglia is at the basis of neurocognitive symptoms observed in neurobrucellosis (8, 24). In the present study, we have evidence that, besides infection, the bystander activation of microglia through inflammatory mediators secreted by *Brucella*-infected astrocytes can also induce such a phenomenon and that IL-6 is essential for the phagocytosis of neurons to take place. Since the use of humanized anti-interleukin-6 receptor antibody (tocilizumab) has been identified as a putative treatment for inflammatory encephalopathies involving neurological symptoms (77–79), the data presented in this paper suggest that the use of such a therapeutic approach might represent a medical strategy to restrict IL-6 signaling, possibly reducing the symptoms associated with neurobrucellosis or other neurological diseases in which the phagocytic activation of microglia is involved.

## Data availability statement

The raw data supporting the conclusions of this article will be made available by the authors, without undue reservation.

## Ethics statement

The animal study was approved by Ethics Committee of Care and Use of Laboratory animals of the School of Medicine, Universidad de Buenos Aires. The study was conducted in accordance with the local legislation and institutional requirements.

## Author contributions

JR: Formal analysis, Investigation, Methodology, Writing – original draft. JS: Formal analysis, Investigation, Methodology, Writing – review & editing. VD: Funding acquisition, Investigation, Writing – review & editing. AR: Formal analysis, Funding acquisition, Investigation, Supervision, Writing – original draft. GG: Conceptualization, Funding acquisition, Investigation, Project administration, Supervision, Writing – review & editing.

## References

[1] Pappas G Akritidis N Bosilkovski M Tsianos E. Brucellosis. N Engl J Med (2005) 352(22):2325–36. doi: 10.1056/NEJMra050570 15930423

[2] Bouza E Garcia de la Torre M Parras F Guerrero A Rodriguez-Creixems M Gobernado J. Brucellar meningitis. Rev Infect Dis (1987) 9(4):810–22. doi: 10.1093/clinids/9.4.810 3326128

[3] McLean DR Russell N Khan MY. Neurobrucellosis: clinical and therapeutic features. Clin Infect diseases: an Off Publ Infect Dis Soc America. (1992) 15(4):582–90. doi: 10.1093/clind/15.4.582 1420670

[4] Nalini A Nagarathna S Rajeshwari S Rose D Veena Kumari HB Nagalingam M. Dementia, peripheral neuropathy, and chronic meningitis in Neurobrucellosis. Indian J Pathol Microbiol (2012) 55(1):128–30. doi: 10.4103/0377-4929.94896 22499327

[5] Shehata GA Abdel-Baky L Rashed H Elamin H. Neuropsychiatric evaluation of patients with brucellosis. J neurovirology. (2010) 16(1):48–55. doi: 10.3109/13550280903586386 20151851

[6] Wallach JC Baldi PC Fossati CA. Clinical and diagnostic aspects of relapsing meningoencephalitis due to Brucella suis. Eur J Clin Microbiol Infect diseases: Off Publ Eur Soc Clin Microbiol (2002) 21(10):760–2. doi: 10.1007/s10096-002-0817-y 12415479

[7] Giambartolomei GH Wallach JC Baldi PC. Neurobrucellosis. In: Encephalitis: Diagnosis and Treatment, vol. 14. . USA: The Egerton Group New York (2008). p. 255–72.

[8] Rodriguez AM Delpino MV Miraglia MC Costa Franco MM Barrionuevo P Dennis VA. Brucella abortus-activated microglia induce neuronal death through primary phagocytosis. Glia. (2017) 65(7):1137–51. doi: 10.1002/glia.23149 28398652

[9] Allen NJ Lyons DA. Glia as architects of central nervous system formation and function. Science. (2018) 362(6411):181–5. doi: 10.1126/science.aat0473 PMC6292669 30309945

[10] Chung WS Allen NJ Eroglu C. Astrocytes control synapse formation, function, and elimination. Cold Spring Harbor Perspect Biol (2015) 7(9):a020370. doi: 10.1101/cshperspect.a020370 PMC4527946 25663667

[11] Fields RD Stevens-Graham B. New insights into neuron-glia communication. Science. (2002) 298(5593):556–62. doi: 10.1126/science.298.5593.556 PMC1226318 12386325

[12] Liddelow SA Barres BA. Reactive astrocytes: production, function, and therapeutic potential. Immunity. (2017) 46(6):957–67. doi: 10.1016/j.immuni.2017.06.006 28636962

[13] Wheeler MA Clark IC Tjon EC Li Z Zandee SEJ Couturier CP. MAFG-driven astrocytes promote CNS inflammation. Nature. (2020) 578(7796):593–9. doi: 10.1038/s41586-020-1999-0 PMC8049843 32051591

[14] Colombo E Farina C. Astrocytes: key regulators of neuroinflammation. Trends Immunol (2016) 37(9):608–20. doi: 10.1016/j.it.2016.06.006 27443914

[15] Prinz M Jung S Priller J. Microglia biology: one century of evolving concepts. Cell. (2019) 179(2):292–311. doi: 10.1016/j.cell.2019.08.053 31585077

[16] Rothhammer V Borucki DM Tjon EC Takenaka MC Chao CC Ardura-Fabregat A. Microglial control of astrocytes in response to microbial metabolites. Nature. (2018) 557(7707):724–8. doi: 10.1038/s41586-018-0119-x PMC6422159 29769726

[17] Vainchtein ID Chin G Cho FS Kelley KW Miller JG Chien EC. Astrocyte-derived interleukin-33 promotes microglial synapse engulfment and neural circuit development. Science. (2018) 359(6381):1269–73. doi: 10.1126/science.aal3589 PMC6070131 29420261

[18] Pfrieger FW Barres BA. Synaptic efficacy enhanced by glial cells in vitro. Science (1997) 277(5332):1684–7. doi: 10.1126/science.277.5332.1684 9287225

[19] Matejuk A Ransohoff RM. Crosstalk between astrocytes and microglia: an overview. Front Immunol (2020) 11:1416. doi: 10.3389/fimmu.2020.01416 32765501 PMC7378357

[20] Garcia Samartino C Delpino MV Pott Godoy C Di Genaro MS Pasquevich KA Zwerdling A. Brucella abortus induces the secretion of proinflammatory mediators from glial cells leading to astrocyte apoptosis. Am J pathology. (2010) 176(3):1323–38. doi: 10.2353/ajpath.2010.090503 PMC2830821 20093491

[21] Miraglia MC Scian R Samartino CG Barrionuevo P Rodriguez AM Ibanez AE. Brucella abortus induces TNF-alpha-dependent astroglial MMP-9 secretion through mitogen-activated protein kinases. J neuroinflammation. (2013) 10:47. doi: 10.1186/1742-2094-10-47 23587438 PMC3637408

[22] Baldi PC Giambartolomei GH. Immunopathology of brucella infection. Recent patents anti-infective Drug discovery. (2013) 8(1):18–26. doi: 10.2174/1574891X11308010005 22812614

[23] Baldi PC Giambartolomei GH. Pathogenesis and pathobiology of zoonotic brucellosis in humans. Rev scientifique technique. (2013) 32(1):117–25. doi: 10.20506/rst.32.1.2192 23837370

[24] Rodriguez AM Delpino MV Miraglia MC Giambartolomei GH. Immune mediators of pathology in neurobrucellosis: from blood to central nervous system. Neuroscience. (2019) 410:264–73. doi: 10.1016/j.neuroscience.2019.05.018 31128159

[25] Liddelow SA Guttenplan KA Clarke LE Bennett FC Bohlen CJ Schirmer L. Neurotoxic reactive astrocytes are induced by activated microglia. Nature. (2017) 541(7638):481–7. doi: 10.1038/nature21029 PMC5404890 28099414

[26] White RE Yin FQ Jakeman LB. TGF-alpha increases astrocyte invasion and promotes axonal growth into the lesion following spinal cord injury in mice. Exp neurology. (2008) 214(1):10–24. doi: 10.1016/j.expneurol.2008.06.012 PMC2895965 18647603

[27] Sanchis P Fernandez-Gayol O Comes G Escrig A Giralt M Palmiter RD. Interleukin-6 derived from the central nervous system may influence the pathogenesis of experimental autoimmune encephalomyelitis in a cell-dependent manner. Cells (2020) 9(2):330. doi: 10.3390/cells9020330 PMC7072597 32023844

[28] Wheeler MA Jaronen M Covacu R Zandee SEJ Scalisi G Rothhammer V. Environmental control of astrocyte pathogenic activities in CNS inflammation. Cell. (2019) 176(3):581–96.e18. doi: 10.1016/j.cell.2018.12.012 30661753 PMC6440749

[29] Savarin C Hinton DR Valentin-Torres A Chen Z Trapp BD Bergmann CC. Astrocyte response to IFN-gamma limits IL-6-mediated microglia activation and progressive autoimmune encephalomyelitis. J neuroinflammation. (2015) 12:79. doi: 10.1186/s12974-015-0293-9 25896970 PMC4410573

[30] Pollak CN Delpino MV Fossati CA Baldi PC. Outer membrane vesicles from Brucella abortus promote bacterial internalization by human monocytes and modulate their innate immune response. PloS One (2012) 7(11):e50214. doi: 10.1371/journal.pone.0050214 23189190 PMC3506553

[31] Rodriguez AM Trotta A Melnyczajko AP Miraglia MC Kim KS Delpino MV. Brucella abortus-Stimulated Platelets Activate Brain Microvascular Endothelial Cells Increasing Cell Transmigration through the Erk1/2 Pathway. Pathogens (2020) 9(9):708. doi: 10.3390/pathogens9090708 PMC7558107 32867217

[32] Harms AS Lee JK Nguyen TA Chang J Ruhn KM Trevino I. Regulation of microglia effector functions by tumor necrosis factor signaling. Glia (2012) 60(2):189–202. doi: 10.1002/glia.21254 21989628 PMC3232308

[33] West PK Viengkhou B Campbell IL Hofer MJ. Microglia responses to interleukin-6 and type I interferons in neuroinflammatory disease. Glia (2019) 67(10):1821–41. doi: 10.1002/glia.23634 31033014

[34] Rothaug M Becker-Pauly C Rose-John S. The role of interleukin-6 signaling in nervous tissue. Biochim Biophys Acta (2016) 1863(6 Pt A):1218–27. doi: 10.1016/j.bbamcr.2016.03.018 27016501

[35] Monif M Reid CA Powell KL Drummond KJ O’Brien TJ Williams DA. Interleukin-1beta has trophic effects in microglia and its release is mediated by P2X7R pore. J neuroinflammation. (2016) 13(1):173. doi: 10.1186/s12974-016-0621-8 27364756 PMC4929731

[36] Taga T Hibi M Hirata Y Yamasaki K Yasukawa K Matsuda T. Interleukin-6 triggers the association of its receptor with a possible signal transducer, gp130. Cell. (1989) 58(3):573–81. doi: 10.1016/0092-8674(89)90438-8 2788034

[37] Hibi M Murakami M Saito M Hirano T Taga T Kishimoto T. Molecular cloning and expression of an IL-6 signal transducer, gp130. Cell. (1990) 63(6):1149–57. doi: 10.1016/0092-8674(90)90411-7 2261637

[38] Rose-John S Heinrich PC. Soluble receptors for cytokines and growth factors: generation and biological function. Biochem J (1994) 300(Pt 2):281–90. doi: 10.1042/bj3000281 PMC1138158 8002928

[39] Jostock T Mullberg J Ozbek S Atreya R Blinn G Voltz N. Soluble gp130 is the natural inhibitor of soluble interleukin-6 receptor transsignaling responses. Eur J Biochem (2001) 268(1):160–7. doi: 10.1046/j.1432-1327.2001.01867.x 11121117

[40] Miraglia MC Rodriguez AM Barrionuevo P Rodriguez J Kim KS Dennis VA. Brucella abortus traverses brain microvascular endothelial cells using infected monocytes as a trojan horse. Front Cell infection Microbiol (2018) 8:200. doi: 10.3389/fcimb.2018.00200 PMC6011031 29963502

[41] Miraglia MC Costa Franco MM Rodriguez AM Bellozi PM Ferrari CC Farias MI. Glial cell-elicited activation of brain microvasculature in response to brucella abortus infection requires ASC inflammasome-dependent IL-1beta production. J Immunol (2016) 196(9):3794–805. doi: 10.4049/jimmunol.1500908 26983788

[42] Celli J de Chastellier C Franchini DM Pizarro-Cerda J Moreno E Gorvel JP. Brucella evades macrophage killing via VirB-dependent sustained interactions with the endoplasmic reticulum. J Exp Med (2003) 198(4):545–56. doi: 10.1084/jem.20030088 PMC2194179 12925673

[43] Barrionuevo P Delpino MV Pozner RG Velasquez LN Cassataro J Giambartolomei GH. Brucella abortus induces intracellular retention of MHC-I molecules in human macrophages down-modulating cytotoxic CD8(+) T cell responses. Cell Microbiol (2013) 15(4):487–502. doi: 10.1111/cmi.12058 23107169

[44] Dolowschiak T Chassin C Ben Mkaddem S Fuchs TM Weiss S Vandewalle A. Potentiation of epithelial innate host responses by intercellular communication. PloS Pathog (2010) 6(11):e1001194. doi: 10.1371/journal.ppat.1001194 21124989 PMC2987820

[45] Copenhaver AM Casson CN Nguyen HT Duda MM Shin S. IL-1R signaling enables bystander cells to overcome bacterial blockade of host protein synthesis. Proc Natl Acad Sci United States America. (2015) 112(24):7557–62. doi: 10.1073/pnas.1501289112 PMC4475993 26034289

[46] Woodward JJ Iavarone AT Portnoy DA. c-di-AMP secreted by intracellular Listeria monocytogenes activates a host type I interferon response. Science. (2010) 328(5986):1703–5. doi: 10.1126/science.1189801 PMC3156580 20508090

[47] Salter MW Stevens B. Microglia emerge as central players in brain disease. Nat Med (2017) 23(9):1018–27. doi: 10.1038/nm.4397 28886007

[48] Farina C Aloisi F Meinl E. Astrocytes are active players in cerebral innate immunity. Trends Immunol (2007) 28(3):138–45. doi: 10.1016/j.it.2007.01.005 17276138

[49] Heyser CJ Masliah E Samimi A Campbell IL Gold LH. Progressive decline in avoidance learning paralleled by inflammatory neurodegeneration in transgenic mice expressing interleukin 6 in the brain. Proc Natl Acad Sci United States America. (1997) 94(4):1500–5. doi: 10.1073/pnas.94.4.1500 PMC19820 9037082

[50] Campbell IL Abraham CR Masliah E Kemper P Inglis JD Oldstone MB. Neurologic disease induced in transgenic mice by cerebral overexpression of interleukin 6. Proc Natl Acad Sci United States America. (1993) 90(21):10061–5. doi: 10.1073/pnas.90.21.10061 PMC47713 7694279

[51] Gyengesi E Rangel A Ullah F Liang H Niedermayer G Asgarov R. Chronic microglial activation in the GFAP-IL6 mouse contributes to age-dependent cerebellar volume loss and impairment in motor function. Front Neurosci (2019) 13:303. doi: 10.3389/fnins.2019.00303 31001075 PMC6456818

[52] Gou X Yuan J Wang H Wang X Xiao J Chen J. IL-6 during influenza-streptococcus pneumoniae co-infected pneumonia-A protector. Front Immunol (2019) 10:3102. doi: 10.3389/fimmu.2019.03102 32038632 PMC6985362

[53] Frisdal E Lesnik P Olivier M Robillard P Chapman MJ Huby T. Interleukin-6 protects human macrophages from cellular cholesterol accumulation and attenuates the proinflammatory response. J Biol Chem (2011) 286(35):30926–36. doi: 10.1074/jbc.M111.264325 PMC3162452 21757719

[54] Chakrabarty P Jansen-West K Beccard A Ceballos-Diaz C Levites Y Verbeeck C. Massive gliosis induced by interleukin-6 suppresses Abeta deposition in *vivo*: evidence against inflammation as a driving force for amyloid deposition. FASEB journal: Off Publ Fed Am Societies Exp Biol (2010) 24(2):548–59. doi: 10.1096/fj.09-141754 PMC3083918 19825975

[55] West PK McCorkindale AN Guennewig B Ashhurst TM Viengkhou B Hayashida E. The cytokines interleukin-6 and interferon-alpha induce distinct microglia phenotypes. J neuroinflammation. (2022) 19(1):96. doi: 10.1186/s12974-022-02441-x 35429976 PMC9013466

[56] Schoeler D Grutzkau A Henz BM Kuchler J Kruger-Krasagakis S. Interleukin-6 enhances whereas tumor necrosis factor alpha and interferons inhibit integrin expression and adhesion of human mast cells to extracellular matrix proteins. J Invest Dermatol (2003) 120(5):795–801. doi: 10.1046/j.1523-1747.2003.12126.x 12713584

[57] Erta M Quintana A Hidalgo J. Interleukin-6, a major cytokine in the central nervous system. Int J Biol Sci (2012) 8(9):1254–66. doi: 10.7150/ijbs.4679 PMC3491449 23136554

[58] Krishnan C Kaplin AI Graber JS Darman JS Kerr DA. Recurrent transverse myelitis following neurobrucellosis: immunologic features and beneficial response to immunosuppression. J neurovirology. (2005) 11(2):225–31. doi: 10.1080/13550280590922801 16036801

[59] Seidel G Pardo CA Newman-Toker D Olivi A Eberhart CG. Neurobrucellosis presenting as leukoencephalopathy: the role of cytotoxic T lymphocytes. Arch Pathol Lab Med (2003) 127(9):e374–7. doi: 10.5858/2003-127-e374-NPALTR 12946213

[60] Trinschek B Lüssi F Haas J Wildemann B Zipp F Wiendl H. Kinetics of IL-6 production defines T effector cell responsiveness to regulatory T cells in multiple sclerosis. PloS One (2013) 8(10):e77634. doi: 10.1371/journal.pone.0077634 24155968 PMC3796502

[61] Ramesh G Alvarez AL Roberts ED Dennis VA Lasater BL Alvarez X. Pathogenesis of Lyme neuroborreliosis: Borrelia burgdorferi lipoproteins induce both proliferation and apoptosis in rhesus monkey astrocytes. Eur J Immunol (2003) 33(9):2539–50. doi: 10.1002/eji.200323872 12938230

[62] Van Wagoner NJ Benveniste EN. Interleukin-6 expression and regulation in astrocytes. J neuroimmunology (1999) 100(1-2):124–39. doi: 10.1016/S0165-5728(99)00187-3 10695723

[63] Marz P Cheng JG Gadient RA Patterson PH Stoyan T Otten U. Sympathetic neurons can produce and respond to interleukin 6. Proc Natl Acad Sci United States America. (1998) 95(6):3251–6. doi: 10.1073/pnas.95.6.3251 PMC19728 9501249

[64] Kummer KK Zeidler M Kalpachidou T Kress M. Role of IL-6 in the regulation of neuronal development, survival and function. Cytokine. (2021) 144:155582. doi: 10.1016/j.cyto.2021.155582 34058569

[65] Garcia-Juarez M Camacho-Morales A. Defining the role of anti- and pro-inflammatory outcomes of interleukin-6 in mental health. Neuroscience. (2022) 492:32–46. doi: 10.1016/j.neuroscience.2022.03.020 35439579

[66] Baune BT Konrad C Grotegerd D Suslow T Birosova E Ohrmann P. Interleukin-6 gene (IL-6): a possible role in brain morphology in the healthy adult brain. J neuroinflammation. (2012) 9:125. doi: 10.1186/1742-2094-9-125 22695063 PMC3464888

[67] Ting EY Yang AC Tsai SJ. Role of interleukin-6 in depressive disorder. Int J Mol Sci (2020) 21(6):2194. doi: 10.3390/ijms21062194 PMC7139933 32235786

[68] Chase KA Cone JJ Rosen C Sharma RP. The value of interleukin 6 as a peripheral diagnostic marker in schizophrenia. BMC Psychiatry (2016) 16:152. doi: 10.1186/s12888-016-0866-x 27206977 PMC4874006

[69] Abdel-Salam OME. The neurotoxic effects of cannabis on brain: review of clinical and experimental data. Mol Sci Applications. (2022) 2:11–23. doi: 10.37394/232023.2022.2.3

[70] Abdel-Salam OME Youness ER Omara AASEA. Oxidative stress and neuronal injury after cannabis and ketamine administration WSEAS Trans Biol Biomedicine (2021) 18:126–35 doi: 10.37394/23208.2021.18.15

[71] Campbell IL Erta M Lim SL Frausto R May U Rose-John S. Trans-signaling is a dominant mechanism for the pathogenic actions of interleukin-6 in the brain. J neuroscience: Off J Soc Neurosci (2014) 34(7):2503–13. doi: 10.1523/JNEUROSCI.2830-13.2014 PMC6802757 24523541

[72] Hsu MP Frausto R Rose-John S Campbell IL. Analysis of IL-6/gp130 family receptor expression reveals that in contrast to astroglia, microglia lack the oncostatin M receptor and functional responses to oncostatin M. Glia. (2015) 63(1):132–41. doi: 10.1002/glia.22739 25103368

[73] Lin HW Levison SW. Context-dependent IL-6 potentiation of interferon- gamma-induced IL-12 secretion and CD40 expression in murine microglia. J neurochemistry. (2009) 111(3):808–18. doi: 10.1111/j.1471-4159.2009.06366.x 19712053

[74] Hirota H Kiyama H Kishimoto T Taga T. Accelerated Nerve Regeneration in Mice by upregulated expression of interleukin (IL) 6 and IL-6 receptor after trauma. J Exp Med (1996) 183(6):2627–34. doi: 10.1084/jem.183.6.2627 PMC2192614 8676083

[75] Chucair-Elliott AJ Conrady C Zheng M Kroll CM Lane TE Carr DJ. Microglia-induced IL-6 protects against neuronal loss following HSV-1 infection of neural progenitor cells. Glia. (2014) 62(9):1418–34. doi: 10.1002/glia.22689 PMC4107000 24807365

[76] Yang P Wen H Ou S Cui J Fan D. IL-6 promotes regeneration and functional recovery after cortical spinal tract injury by reactivating intrinsic growth program of neurons and enhancing synapse formation. Exp neurology. (2012) 236(1):19–27. doi: 10.1016/j.expneurol.2012.03.019 22504113

[77] Muccioli L Pensato U Cani I Guerra L Provini F Bordin G. COVID-19-related encephalopathy presenting with aphasia resolving following tocilizumab treatment. J neuroimmunology. (2020) 349:577400. doi: 10.1016/j.jneuroim.2020.577400 33032013 PMC7513756

[78] Borah P Deb PK Chandrasekaran B Goyal M Bansal M Hussain S. Neurological consequences of SARS-CoV-2 infection and concurrence of treatment-induced neuropsychiatric adverse events in COVID-19 patients: navigating the uncharted. Front Mol biosciences. (2021) 8:627723. doi: 10.3389/fmolb.2021.627723 PMC7930836 33681293

[79] Liu L Liu S Guan W Zhang L. Efficacy of tocilizumab for psychiatric symptoms associated with relapsing polychondritis: the first case report and review of the literature. Rheumatol Int (2016) 36(8):1185–9. doi: 10.1007/s00296-016-3509-0 27260262

